# The Burden of Modifiable Cardiovascular Risk Factors in a Population of Central Italy: A Pilot Study

**DOI:** 10.3390/healthcare11101473

**Published:** 2023-05-18

**Authors:** Emma Altobelli, Francesca Marzi, Paolo Matteo Angeletti, Francesca Galassi, Antonello Karim Guercache, Valerio Filippo Profeta, Anna Maria Angelone

**Affiliations:** 1 Department of Life, Public Health and Environmental Sciences, University of L’Aquila, 67100 L’Aquila, Italy; francesca.marzi@univaq.it (F.M.); paolomatteoangeletti@gmail.com (P.M.A.); francesca.galassi@graduate.univaq.it (F.G.); antonellokarim.guercache@graduate.univaq.it (A.K.G.); annamaria.angelone@univaq.it (A.M.A.); 2 Department of Territorial Assistance, ASL Teramo, 74100 Teramo, Italy; valerio.profeta@aslteramo.it

**Keywords:** cardiovascular screening, lifestyle, Italy, risk factor

## Abstract

Background: By the mid-20th century, cardiovascular disease (CVD) had become an important cause of mortality and morbidity in developed countries. The aim was to set up a pilot study to screen citizens aged 45–59 in order to identify modifiable risk factors (RFs). Methods: Our study was conducted from February 2019 to February 2020 on citizens of a population of central Italy, aged 45–59, contacted by letter. The variables evaluated were lipid profile, glycemia, anthropometric parameters, lifestyle and utility of screening. Results: It is important to underline that from the comparison with Italian national data, our results showed that blood pressure and lipid profile had better values. On the contrary, there were some alarm bells: a high percentage (57%) of smokers (63.9 in men and 37.1 in women), a sedentary lifestyle (24.5%), and a significantly higher waist circumference than the reference cut-offs for both men and women. The organization of the screening was considered excellent by 56.3% of women and 48.4% of men, and good by 37.5% of women and 46.5% of men. Conclusions: Our study provides a picture to stakeholders of the state of the health of citizens in the area under study, in the immediate pre-pandemic period; however, it is important to underline that their state of health may be modified after the pandemic period. Furthermore, cardiovascular (CV) screening was perceived by the citizens to be important for health care.

## 1. Introduction

The progressive increase in chronic pathologies is becoming not only a health emergency, but also a social emergency. Of note are cardiovascular diseases (CVD), which became the leading cause of death and morbidity in developed Western countries in the mid-20th century [1]. Cardiovascular disease prevention programs have resulted in a 50% mortality reduction thanks to the removal of risk factors and a 40% improvement in therapies [2]. The Global Burden of Disease Study has shown that in the last 30 years, there has been a profound change in the prevalence and global impact of risk factors, due to the phenomena of epidemiological transition on a global scale [3]. Most cases of CVD are associated with a handful of common, modifiable risk factors [4]. The most important way to prevent CVD is to promote a healthy lifestyle. Therefore, the management of known modifiable risk factors such as smoking, alcohol, a sedentary lifestyle, overweight and obesity should be highlighted [4]. Control of the lipid profile and glycemia i also recommended [5]. Arnett et al. [6] have indicated the top 10 take-home messages for primary prevention of CVD as standard for guiding management of risk factors.

In Italy in 2015, there were 240,000 deaths from CVD, making up about 37% of total deaths, representing an increase of 8.8% compared to 2014 [7]. The data reported by the Heart Project of the Istituto Superiore di Sanità (2016) showed that the most frequent risk factors in the 35–74 age group are LDL hyperlipidemia (67.5% in males and 68.7% in females) and hypercholesterolemia in 65.8% of males and 69.3% of females; arterial hypertension, overweight and physical inactivity follow [8]. The Italian National Prevention Plan (NPP) (2014–2018, extended to 2019) [9], among its main objectives, envisaged a reduction in the preventable and avoidable burden of morbidity, mortality and disability from non-communicable diseases, among which CVDs were of particular relevance. The early identification and integrated assessment of citizens at risk of CVD is an indispensable strategy today, with subsequent systematic management capable of increasing people’s empowerment for the adoption of better lifestyles, and an of delivering an adequate therapeutic approach.

An Abruzzo regional strategy was to implement population-based screening for the early identification of individuals at risk of CVD. In this context, the aims of our study were (i) to set up a pilot study to screen citizens aged 45–59, an age group that rarely turns to a general practitioner to check their health status, in order to identify the main modifiable risk factors to program preventive strategies; (ii) to compare their blood pressure parameters, lipid profile, glycemia, BMI and waist circumference with those of the Italian national data; and (iii) to evaluate their degree of satisfaction with the screening. 

## 2. Materials and Methods

**Figure 1:**
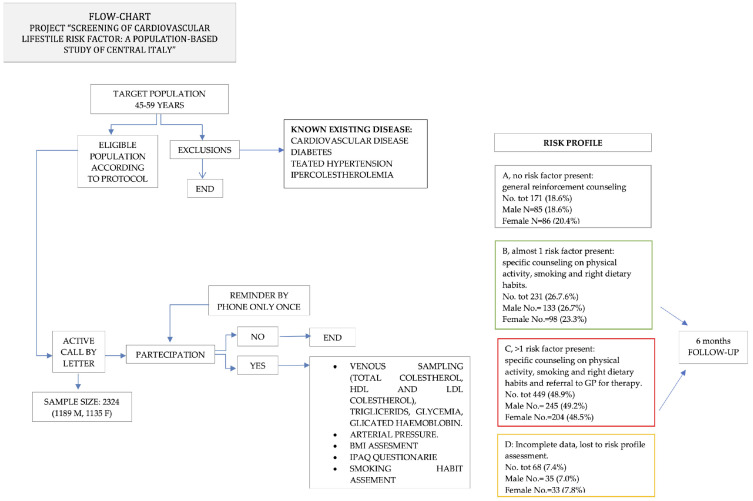
Study flowchart. Informative material was also delivered, and a 6-month check-up was recommended to assess the impact of counseling.

A screening of primary cardiovascular disease prevention was conducted from 1 February 2019 to 28 February 2020 in the Teramo province, central Italy, on a population aged 45–59 years. The target population residing in Teramo province on 1 January 2019 consisted of 72,181 citizens, of which 35,463 were males and 36,718 were females (Figure 1). This screening was approved by the Abruzzo region’s Department of Public Health [10], and it was free of charge for all participants. In order to estimate the sampling dimension (n = 2324), the following parameters were used: sample error E = 2 and 1-alpha = 0.95. The exclusion criteria were cardiovascular diseases, diabetes mellitus, hypercholesterolemia and hypertension under treatment.

The citizens were contacted by a letter explaining the scope of the screening and the diagnostic test involved (venous blood sampling for total cholesterol, HDL, LDL, triglycerides and glycemia); this was followed-up by a telephone call a few days before the appointment of the clinical visit. The study flow-chart is presented in Figure 1. Citizens that chose to participate signed an informed consent form. A questionnaire structured into three parts, validated through a pilot study, was administered during the clinical visit, and some information was collected. The first part consisted of sociodemographic, anamnestic, clinical and laboratoristic variables, the second of anthropometric parameters (weight, height, BMI), lifestyle (smoking), and the third of questions related to variables on the utility of the screening, its organization, the level of service offered, the clarity of the information provided, and the need for information on cardiovascular prevention. In addition, blood pressure parameters, lipid profile, glycemia, BMI and waist circumference were compared with Italian national data, referring to a healthy population according to age and gender [11].

To evaluate physical activity, we used an IPAQ questionnaire short form [12]. Physical activity was classified into three groups of weekly moderate physical activity: low, moderate or high, according to Guidelines for the data processing and analysis of the International Physical Activity Questionnaire (IPAQ) [13]. Following counseling and clinical laboratory results, the subjects were classified into four groups, for which different follow-up paths were chosen (Figure 1):

- group A. Patients who had an adequate lifestyle (no smoking habit, intense or moderate physical activity and a varied diet) and who had normal blood and anthropometric parameters. These patients were given a simple “reinforcement” counseling to encourage them to continue with their adequate lifestyle, to carry out regular moderate physical activities, and to attend a further check-up after 2 years;
- group B. Patients with behavioral risk factors such as cigarette smoking or low physical activity, but with normal blood and clinical parameters. These patients were offered individual counseling with information on effective methodologies for quitting smoking, or advice on how to carry out adequate physical activity and maintain a balanced diet.
- group C. Patients with altered clinical blood parameters, incorrect or inadequate lifestyles and abnormal blood and clinical parameters. These patients were sent to their general practitioner (GP) for further diagnostic tests. The GP would then propose a normalization of these parameters, favoring lifestyle intervention, or, in more serious cases, a pharmacological approach;
- group D. Patients who were not eligible according to the exclusion criteria, or who had not completed all the examinations required by the screening (medical examination and measurement of blood parameters). They were automatically left out of the program.

The associations among variables collected were analyzed using a chi-squared test, a *t*-test and a Kruskal–Wallis where appropriate. All analyses were conducted using the software SAS/STAT (version 15.3).

## 3. Results

During the period April 2019–February 2020, 2324 citizens were invited by letter. A total of 919 citizens (39.6%) participated in the screening, of which 498 were males and 421 were females. The study participants had a mean age of 54.6 years ± 4.2 (males 53.6 ± 4.2; females 55.7 ± 3.9), and therefore were comparable in age.

### 3.1. Clinical Parameters

Our results showed that the systolic and diastolic blood pressure values were higher in males than in females, respectively (126.1 ± 15.2 vs. 121.4 ± 17.0, *p* < 0.001 and 81.1 ± 9.3 vs. 76.7 ± 9.8; *p* < 0.001). On the contrary, the total and LDL cholesterol values did not show statistically significant differences (204 ± 38.0 vs. 209.3 ± 37.3 mg/dL *p* = 0.081; 133.2 ± 34.4 vs. 132.2 ±32.7 mg/dL; *p* = 0.741); on the other hand, HDL cholesterol values were higher in women (62.7 ± 14.9 vs. 52.2 ± 12.5 mg/dL *p* < 0.001). Triglycerides were higher in men than in women (129.1 ± 91.6 vs. 105.4 ± 62.8 mg/dL; *p* < 0.001), as were blood glucose values (98.5 ± 19.5 vs. 94.1 ± 20.7 mg/dL; *p* = 0.001) and the total cholesterol/HDL ratio (4.1 ± 1.1. vs. 3.5 ± 1.1 *p* < 0.001). In males, the mean BMI was 27.3 ± 4.0 kg/m^2^, while in females, it was 25.8 ± 5.1 kg/m^2^ (*p* < 0.0001).

Table 1 shows our results relating to the blood pressure parameters, lipid profile, glycemia, BMI and waist circumference, stratified by gender and by age group (45–54 years and 55–59 years), compared with the Italian national data (IND). Compared to IND, statistically significant differences were found in females aged 45–54 for blood pressure, total cholesterol, and LDL cholesterol. In the age group of women 55–59 years, triglycerides and BMI values were also statistically lower than the national data (Table 1). Similarly, for males aged 45–54, there were statistically significant differences in blood pressure, total cholesterol, LDL cholesterol, triglycerides and glycemic values. On the other hand, for those aged 55–59 years, only blood pressure, total cholesterol and glycemic values were statistically significant. On the contrary, it is important to underline that the waist circumference values were higher than those of Italian data (101 ± 10.6 vs. 97.9 ± 10.9; *p* = 0.006).

### 3.2. Lifestyle

**Figure 2:**
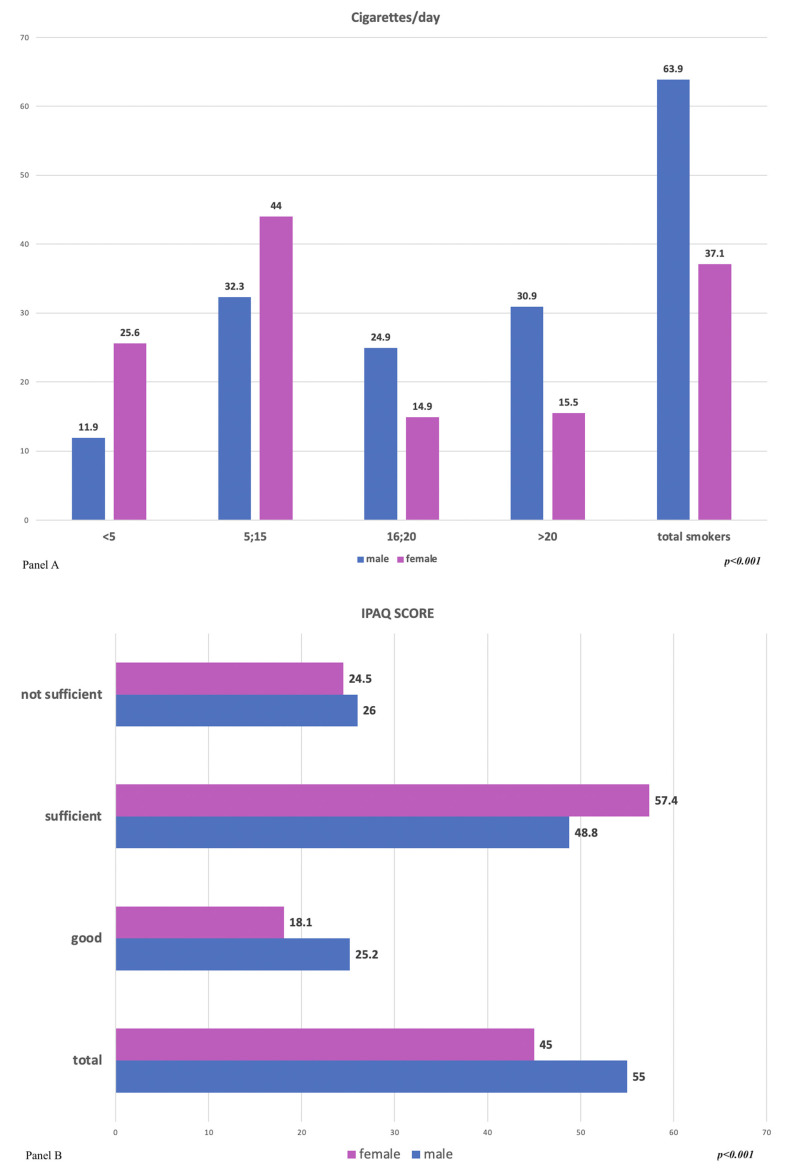
Panel (**A**): Cigarettes/day in males and females; Panel (**B**): IPAQ score results in males and females.

Regarding smoking, 52.7% (n = 475) of the interviewees stated that they had smoked at least once in their life. Of these, 4.7% (n = 234) currently smoke, while 50.3% (n = 237) have quit for at least 1 year; some 47.3% (n = 427) said they had never smoked (Figure 2, panel A).

With respect to gender, smoking was more common in men than in women (63.9% vs. 37.1%, *p* < 0.001). Furthermore, men smoked more than women (Figure 2, panel A).

The results of the IPAQ questionnaire showed a higher very active level of physical activity in males (25.2%) then in females (18.1%), a higher sufficiently active level (57.4%) in females than in males (48.8%), and a higher insufficient level of activity in males (26.0%) than in females (24.6%) (*p* < 0.027) (Figure 2, panel B). 

### 3.3. Waist Circumference

The mean value of waist circumference in males was (n = 476) 100.3 ± 11.6 cm, while in females (n = 395), it was 88.9 ± 12.7 cm. Both averages were higher than the values established by the moderate risk guidelines (<94 cm in males and <80 cm in females).

**Figure 3:**
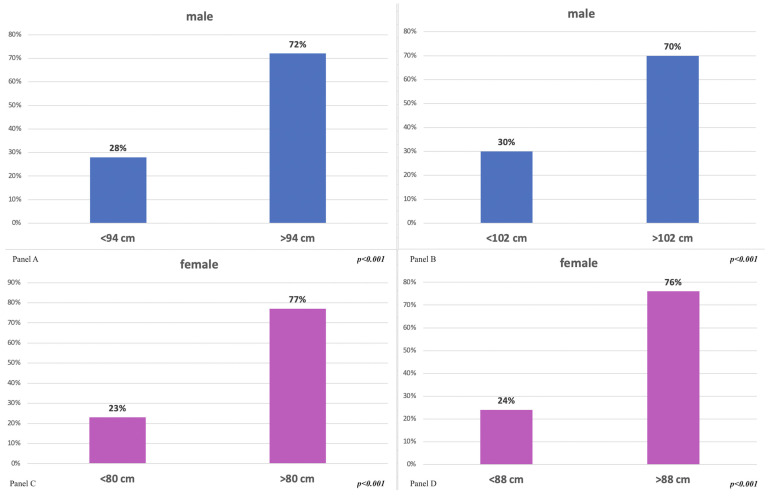
Panel (**A**): waist circumference in males, medium and high-risk. Panel (**B**): waist circumference in males, high-risk; Panel (**C**): waist circumference in females, medium and high-risk. Panel (**D**): waist circumference in females, high-risk.

Some 72.1% of males (n = 343) presented a waist circumference ≥ 94 cm, while 27.9% showed a circumference < 94 (n = 133). Some 77.5% of females (n = 306) presented a value ≥ 80 cm, while 22.5% (n = 89) showed a circumference < 80. It is important to underline that 37.6% (n = 179) of males had a high-risk waist circumference, i.e., >102 cm, and 45.1% (n = 178) of females had a high-risk waist circumference, i.e., >88 cm (Figure 3). 

### 3.4. BMI

Table 2 shows the five BMI classes. Compared to gender, there was an overall statistically different distribution between genders (*p* < 0.001). For the BMI in the normal range (18.5–24.9), females were found to be in greater numbers (44.4 vs. 27.8%) than males. 

Instead, in the overweight range (BMI 25.0–29.9), males showed a higher frequency than females (45.1% vs. 33.6%); this trend was also confirmed for first-degree obesity (19.3% vs. 12.2%). In the underweight and in the severely obese range, no differences were found between sexes.

The distribution of BMI and LDL cholesterol values with respect to age groups did not show statistically significant differences (*p* = 0.478, *p* = 0.155); conversely, the frequency of physical activity was significantly different (*p* < 0.001). It can be seen that high levels of physical activity were more frequent in the normal weight range, while low or moderate levels of activity were present in the overweight range. In the BMI range of 30.0–34.9, there was a higher frequency of low physical activity. The distribution of triglycerides with respect to BMI was also statistically significant (*p* < 0.001), even if the frequencies were not relevant.

### 3.5. Degree of Satisfaction Concerning the Quality of the Assistance and Cardiovascular Risk Perceived

Finally, in screened subjects, questions were asked relating to (i) the frequency of visits to their general practitioner (GP); (ii) the information sources on cardiovascular (CV) risk; (iii) their perception of the organization of the screening; (iv) the utility of the screening; and (v) the clarity of the information obtained following the screening on their own CV risk.

With respect to the organization of the screening, this was considered excellent by 56.3% of women and 48.4% of men, and good by 37.5% of women and 46.5% of men (*p* = 0.046) (Table 3).

## 4. Discussion

The control of cardiovascular diseases is the goal of the WHO Global Action Plan for the Prevention and Control of NCDs 2013–2020, through combined strategies that include a life course approach, which consider the empowerment of the individual and the community [14]. These strategies should provide an integrated approach in which access to healthcare resources is guaranteed to all citizens, breaking down any social inequality [14]. It is known that making lifestyle changes (nutrition, smoking habits, physical activity, alcohol, etc.) can radically change not only one’s cardiovascular risk profile, but also the metabolic and oncological health of the individual [15,16,17,18].

In Italy, in the period 1980–2000, mortality from cardiovascular diseases decreased from 267.1 to 141.3 per 100,000 inhabitants in men aged 25 to 84, and from 161.3 to 78.8 per 100,000 inhabitants in the general population [19]. This decrease in mortality is most likely attributable to the prevention and control of modifiable risk factors. In particular, a reduction in systolic blood pressure by at least 5.5 mmHg, a reduction in total cholesterol by at least 0.35 mmol/L, a 4% prevalence of smoking, and 10% of people being physically active may significantly contribute to the reduction in mortality [19].

A recent work by Han et al. [20] has shown that in many European cities, there has been an increase in average BMI and waist circumference (WC). This indicates how essential it is to plan correct CV risk control and prevention strategies. Palmieri et al. [19] state that a 0.1% increase in diabetes mellitus in the general population can lead to about 1000 more deaths, and even a small increase in BMI can cause an increase of about 250 deaths. These data are confirmed by a large work conducted on 61 prospective studies; 1 mmol less cholesterol reduces the risk of cardiovascular mortality in all ages [21], reaffirming the fundamental importance of primary prevention in the most affected risk classes.

It is important to underline that from the comparison with the Italian national data, our results show that some metabolic parameters considered to be of cardiovascular risk, such as blood pressure and total cholesterol, have better values. These results are consistent with what has already emerged from the Heart Project study, which highlighted interregional differences in hypercholesterolemia [22].

On the contrary, there are some alarm bells, such as the high percentage of smokers (63.9 in men and 37.1 in women, 57% of the total) and a sedentary lifestyle; in fact, 24.5% of females and 26.0% of men do not practice sufficient physical activity (Figure 2). Furthermore, in our sample, the waist circumference, a fundamental parameter in the calculation of CV risk, which correlates with visceral fat [23], was significantly higher in our results than the reference cut-offs for men and women. Additionally, in this case, significant inter-regional differences can be identified in Italy [24].

Recent evidence on non-alcoholic fatty liver disease suggests a link between the latter with cardiovascular risk; an evaluation of liver ultrasound imaging could be of help in defining the populations most at risk [25,26].

Our analysis shows a gender gap regarding the distribution of risk factors in males, who therefore need more attention. However, it should not be forgotten that in the menopausal period, the risk affecting men and women tends to be equal [27].

Other important aspects to be taken into consideration in our study have highlighted the importance of citizens having information on health care from GPs, through a “doctor–patient relationship”, as well as through information campaigns. It is known that adherence to preventive programs improves with good doctor–patient communication [28]. 

In fact, the importance of CV screening was perceived by citizens as an important tool for CV prevention and health care.

## 5. Conclusions

Our study highlights some important aspects. The first provides stakeholders with a picture of the state of the health of citizens in the area under study, in the immediate pre-pandemic period. The second conclusion is that the importance of CV screening was perceived by citizens as an important tool for CV prevention and health care; third, this study highlights the satisfactory state of health in the population studied in the pre-pandemic period, which may be different in the post-pandemic period, thereby suggesting the planning of targeted interventions on the age groups of the population most at risk. The capacity for early intervention through primary and secondary prevention would allow for considerable economic savings, also considering the fact that measuring the state of the health of communities represents a fundamental tool for planning correct resource allocation for the health service.

## Figures and Tables

**Table 1:** Comparison between our samples and the National Heart Project [11] regarding means of parameters.

	Females 45–54 Years					Females 55–59 Years				
	Teramo Province Responders (No. = 135)		Italian National Data [11] (No. = 1025)			Teramo Province Responders (No. = 286)		Italian National Data [11] (No. = 1025)		
**Parameters**	**Mean**	**s.d.**	**Mean**	**s.d.**	***p***	**Mean**	**s.d.**	**Mean**	**s.d.**	***p***
**SBP (mmHg)**	117.2	13.5	122.4	15.9	0.000	123.8	18.0	132.5	17.8	0.000
**DBP (mmHg)**	74.8	8.0	79.3	9.3	0.000	77.8	10.4	81.4	9.5	0.000
**Cholesterol (total mg/dL)**	206.2	33.0	219.7	41.7	0.000	210.8	38.7	229.4	42.3	0.000
**HDL Cholesterol (mg/dL)**	64.5	15.4	62.8	14.7	0.218	62.0	14.6	61.8	14.8	0.840
**Triglycerides (mg/dL)**	94.7	44.6	101.1	54.4	0.200	101.9	68.8	116.2	56.8	0.000
**LDL Cholesterol (mg/dL)**	125.3	28.7	136.6	36.0	0.000	135.7	33.8	144.5	37.7	0.000
**Glycemia (mg/dL)**	88.9	9.2	91.4	17.9	0.053	96.4	23.7	98.1	26.4	0.327
**BMI (Kg/m^2^)**	25.3	4.6	26.4	5.4	0.108	26.0	5.3	28.2	5.6	0.000
**Waist circunference (cm)**	87.4	11.4	85.1	13.1	0.057	89.8	13.3	89.5	13.0	0.732
	**Males 45–54 years**					**Males 55–64 years**				
	**Teramo Province Responders (No. = 256)**		**Italian National Data [11] (No. = 1025)**			**Teramo Province Responders (No. = 242)**		**Italian National Data [11] (No. = 1025)**		
**SBP (mmHg)**	124.2	15.5	130.4	15.7	0.000	128.5	14.6	136.5	17.8	0.000
**DBP (mmHg)**	80.3	9.7	86.3	10.0	0.000	82.2	8.7	85.5	10.1	0.000
**Cholesterol (total mg/dL)**	207.1	37.5	217.0	42.9	0.000	202.5	38.5	212.4	43.7	0.002
**HDL Cholesterol (mg/dL)**	54.7	12.7	49.9	11.7	0.000	51.5	12.1	50.9	12.5	0.512
**Triglycerides (mg/dL)**	126.0	95.3	143.8	94.9	0.007	133.4	90.0	138.8	83.8	0.388
**LDL Cholesterol (mg/dL)**	133.7	33.0	139.0	36.5	0.034	133.3	35.8	134.0	38.3	0.802
**Glycemia (mg/dL)**	96.1	19.8	99.9	22.1	0.012	101.3	19.2	105.5	26.3	0.024
**BMI (Kg/m^2^)**	27.2	4.1	27.7	4.1	0.081	27.6	3.9	28.0	3.9	0.164
**Waist circunference (cm)**	99.8	12.2	100.2	8.2	0.531	101.1	10.6	97.9	10.9	0.006

s.d. = standard deviation, SBP = systolic blood pressure, DBP = diastolic blood pressure, HDL = high-density lipoprotein, LDL = low-density lipoprotein, BMI = BODY MASS INDEX.

**Table 2:** BMI association according to gender, age groups, physical activity and lipid profile.

BMI	No. (%) Underweight < 18.5	No. (%) Normal Weight 18.5–24.9	No. (%) Overweight 25–29.9	No. (%) Obesity I Grade 30–34.9	No. (%) Obesity II Grade 35–39.9	No. (%) Severe Obesity ≥ 40	Total Responders
Gender							
**Male**	18 (3.7)	135 (27.8)	219 (45.1)	94 (19.3)	4 (0.8)	16 (3.3)	486
**Female**	22 (5.3)	185 (44.4)	140 (33.6)	51 (12.2)	3 (0.7)	16 (3.4)	417
**Total**	40 (4.4)	320 (35.4)	359 (39.8)	145 (16.1)	7 (0.8)	32 (3.5)	903
***p* < 0.0001**							
**Age groups**							
**45–54**	14 (3.7)	140 (37.0)	147 (38.9)	62 (16.4)	5 (1.3)	10 (2.7)	378
**55–59**	16 (3.2)	174 (34.7)	206 (41.1)	82 (16.4)	2 (0.4)	21 (4.2)	501
**Total**	30 (3.4)	314 (35.7)	353 (40.2)	144 (16.4)	7 (0.8)	31 (3.5)	879
***p* = 0.478**							
**Physical activity**							
**Low**	3 (1.6)	48 (25.4)	76 (40.2)	50 (26.4)	3 (1.6)	9 (4.8)	189
**Moderate**	12 (3.1)	155 (39.3)	167 (42.4)	47 (11.9)	2 (0.5)	11 (2.8)	394
**High**	9 (5.4)	67 (40.1)	64 (38.3)	23 (13.8)	2 (1.2)	2 (1.2)	167
**Total**	24 (3.2)	270 (36.0)	307 (40.9)	120 (16.0)	7 (1.0)	22 (2.9)	750
***p* < 0.0001**							
**LDL**							
**No. (%) < 115**	15 (39.5)	109 (36.6)	96 (28.6)	36 (26.1)	2 (40.0)	9 (32.1)	267
**N (%) ≥ 115**	23 (60.5)	189 (63.4)	240 (71.4)	102 (73.9)	3 (60.0)	19 (67.9)	576
**Total**	38	298	336	138	5	28	843
***p* = 0.155**							
**Triglycerides**							
**No. (%) < 150**	34 (89.5)	275 (92.0)	267 (78.5)	91 (65.0)	6 (100.0)	20 (71.4)	693 (81.4)
**No. (%) ≥ 150**	4 (10.5)	24 (8.0)	73 (21.5)	49 (35.0)	0 (0.0)	8 (28.6)	158 (18.6)
**Total**	38	299	340	140	6	28	851
***p* < 0.0001**							

**Table 3:** Degree of satisfaction concerning the quality of the assistance perceived.

	No. (%) Males	No. (%) Females	Total Responders
Routine visit at to a general practitioner: every			
15 days	22 (5.4)	14 (4.1)	36
30 days	81 (19.9)	83 (24.2)	164
2–4 months	156 (38.3)	129 (37.6)	285
1 year	148 (36.4)	117 (34.1)	265
Total	407	343	750
			*p* = 0.468
**Patient’s opinion of the best information source**			
Pharmacist	2 (0.5)	6 (1.8)	8
Health personnel	82 (20.3)	71 (20.8)	153
General practitioner	114 (28.2)	93 (27.2)	207
Information campaigns	149 (36.9)	85 (24.8)	234
Newspapers	10 (2.5)	10 (2.9)	20
Television	17 (4.2)	22 (6.4)	39
Internet Social Network	12 (3.0)	14 (4.1)	26
Friends and family	18 (4.4)	41 (12.0)	59
**Total**	404	342	746
			***p* = 0.003**
**Organization screening perceived**			
Very good	196 (48.5)	192 (56.3)	388
Good	188 (46.5)	128 (37.5)	316
Sufficient	20 (5.0)	21 (6.2)	41
**Total**	**404**	**341**	**745**
			***p* = 0.0458**
**Utility screening perceived**			
Very good	218 (54.4)	199 (58.7)	417
Good	171 (42.6)	122 (36.0)	293
Sufficient	12 (3.0)	18 (5.3)	30
**Total**	**401**	**339**	**740**
			*p* = 0.0783
**Patient’s opinion of doctor’s explanation of cardiovascular risk**			
Very good	248 (61.8)	224 (66.7)	472
Good	148 (36.9)	105 (31.2)	253
Sufficient	5 (1.3)	7 (2.1)	12
**Total**	401	336	**737**
			*p* = 0.2071

## Data Availability

Not applicable.
